# The relationship between psychological resilience, neuroticism, attentional bias, and depressive symptoms in college Chinese students

**DOI:** 10.3389/fpsyg.2022.884016

**Published:** 2022-10-20

**Authors:** Mengmei Wang, Jiangbo Li, Guoli Yan, Tong Lei, Wei Rong, Ling Sun

**Affiliations:** ^1^Department of Child and Adolescent Psychology, Tianjin Anding Hospital, Mental Health Center of Tianjin Medical University, Tianjin, China; ^2^Department of Clinical Psychology, Wuhu Hospital Affiliated to East China Normal University, Wuhu, China; ^3^Sichuan Provincial Center for Mental Health, Sichuan Academy of Medical Sciences and Sichuan Provincial People’s Hospital, Chengdu, China; ^4^People’s Hospital of Jianyang City, Chengdu, China

**Keywords:** college freshmen, resilience, attentional bias, depression, neuroticism

## Abstract

In recent years, the incidence of depressive symptoms among Chinese college students has been increasing. Studies have shown that depressive symptoms are related to a variety of psychosocial factors, among which neuroticism, resilience, and attention bias are most notably related, but the correlation among the three is not clear. This study aimed to investigate the influence mechanisms of different degrees of resilience, attentional bias, and neuroticism in the formation of depressive symptoms. The college freshmen of this study were selected through stratified multi-stage cluster sampling. Students provided informed consent and then completed a general situation questionnaire and four scales: the Chinese version of the Connor–Davidson Resilience Scale, the Attention to Positive and Negative Information Scale, the Eysenck Personality Questionnaire, and the Zung Self-Rating Depressive Symptoms Scale. In total, 1,493 freshmen participated in the research group. Our results showed that low resilience, negative attention bias, and high neuroticism jointly increased the risk of depressive symptoms. There is a significant correlation between these three factors and depressive symptoms. Additionally, strength, tenacity, and attention bias all had more significant effects on the occurrence of depressive symptoms. These findings indicate that there may be an important psychological mechanism for the occurrence, development, and poor prognosis of depressive symptoms.

## Introduction

Mental health problems among college students in recent years have been a significant concern for society. According to a meta-analysis of 84 studies involving 1,292,811 college students, the detection rate of depressive symptoms among college students in China was 26% (Luo et al., 2021). The main symptoms of depression are depressed mood, decreased interest, and slow thinking (Fan, 1993), and they can have a serious negative impact on people’s lives (Luo et al., 2021). Depression results from the interaction between individual genes and the environment (Garber and Rao, 2014). Specifically, depressive symptoms are related to negative life events (Chen et al., 2013), interpersonal relationships (Dunn et al., 2011; Yang and Chen, 2015), personality dimension, parenting style, and family environment (Assche et al., 2017). A previous study has shown an increased risk of depression among Chinese college students during the COVID-19 pandemic (Zhang et al., 2021). At the same time, some scholars believe that the high detection rate of depressive symptoms among Chinese college students is related to professional satisfaction, employment prospects, personality, family, ethnicity, physical, academic performance, and interpersonal relationships (Yang and Chen, 2015; Wang M. et al., 2020; Feng et al., 2022). These are the important factors that affect emotions; however, why do some people become depressed while others are not affected by the same factors? Some researchers believe that one crucial factor that determines depressive symptoms is resilience, also known as “mental elasticity” or “recovery power,” which is the psychological characteristic that someone possesses to solve problems and adapt to new situations with a positive mindset (Luthar et al., 2000). Studies have shown that depressive symptoms are associated with poor psychological resilience (Wang C. X. et al., 2020). Resilience has a buffering effect on people when they are faced with unexpected incidents in life and can reduce the symptoms of depression and anxiety (Ye et al., 2016; Sheerin et al., 2018). According to recent research, people with high resilience and a positive attitude toward life have lower levels of anxious and depressive symptoms (Song et al., 2021). The resilience of college students has a negative correlation with the extent of depressive symptoms, and high resilience has a preventive effect on these symptoms (Wei, 2019; Xu et al., 2021). Resilience has both short-term and long-term protective effects on college students (Wu et al., 2020). Guo et al. (2020) found that teacher support enhances mental well-being by decreasing negative emotions and then increasing resilience, meaning that the mediating effect of resilience between teacher support and mental well-being was relatively larger. Cognitive bias is believed to be related to various emotional disorders in human relationships, such as anxiety and depression (Taylor and John, 2004). Cognitive bias can be divided into different categories, including attention bias, interpretation bias, memory bias, and executive control bias (Joormann and Gotlib, 2010; Koster et al., 2011; Murrough et al., 2011). Attention is the first step in the cognitive process and plays a very important role. Studies on attentional bias have shown that depressive symptoms can be alleviated if attentional bias on positivity is enhanced (Dai et al., 2019). Meanwhile, improving attentional control also promotes resilient traits (Schäfer et al., 2015).

Neuroticism is a pattern of negative emotional responses to setbacks, failures, and dangers (Lahey, 2009). One study found that neuroticism significantly predicted depressive mood (Pereira-Morales et al., 2019). Kendler et al. (2004) found that high neuroticism contributes to a high overall risk of an individual’s severe depressive symptoms. Studies have shown that people with high neuroticism are more susceptible to negative stimuli (Qin and Hi, 2008). These studies suggest that resilience, attention bias, and neuroticism may be closely related to depressive symptoms.

However, it is not clear which of the three traits plays a more significant role in depression. Thus, in this study, we aimed to determine the influence of neuroticism, attentional bias, and different degrees of resilience on the occurrence of depressive symptoms, in the hopes that we may understand their different roles and the psychological mechanism of depressive symptoms. We hypothesized that neuroticism, attention bias, and resilience are related to the occurrence of depressive symptoms. We also predicted that neuroticism and negative attention bias may have a greater impact on depressive symptoms under the condition of low resilience. We investigated a large sample of college freshmen to verify our hypothesis and build a foundation for research on the psychological mechanism of the production of depressive symptoms and the prevention and treatment strategy for depression in college students.

## Materials and methods

### Participants and procedure

This study was conducted between February and March 2021. The research data were collected through the Questionnaire Star online system. Participants were selected through stratified multi-stage cluster sampling. First, five universities were randomly selected from a larger group, that is, four from 60 universities in Tianjin and one from 20 universities in Ningxia. In the second stage, we randomly selected a major in each school to recruit students from. A total of 1,698 college freshmen were selected. The school tutor sent the survey link to each student by WeChat. The students read the informed consent form on their cell phones and agreed to participate in the study by completing the form. A total of 1,493 valid questionnaires were collected, representing 87.9% of the total number. There were 568 men and 925 women representing 38 and 62% in the research group, respectively. The average age of the participants was 18.92 ± 0.984 years. The study was approved by the Ethical Committee of the Tianjin Anding Hospital.

### Instruments

#### Demographic information

Participants first completed a demographic information survey form that included age, gender, residence, major, and grade point average.

#### Connor–Davidson resilience scale

The Chinese version of the Connor–Davidson Resilience Scale (CD-RISC) was revised by Nan Xiao and Jianxin Zhang in 2007. The scale has 25 items covering three factors: tenacity (11–23), strength (1, 5, 7–10, 24–25), and optimism (2–4, 6). The scale is scored on a 5-point scale, with 0 meaning “never,” and 4 meaning “always.” The higher a participant’s score, the higher their level of resilience. The sum of the items corresponding to each factor is the factor score. The total scale score is the sum of all items. The original Chinese version of the CD-RISC has a high internal consistency, with Cronbach’s α = 0.91 (Yu and Zhang, 2007).

#### Attention to positive and negative information scale

The Attention to Positive and Negative Information Scale (APNI) was used to assess positive and negative information attentional biases. The APNI was first developed by Noguchi in 2006 and translated and revised into Chinese by Dai et al. (2015). The scale had 22 items, divided into two separate scales of attention to positive information (API; 1, 6, 7, 9, 11, 13–18, 20) and attention to negative information (ANI; 2–5, 8, 10, 12, 19, 21, 22), and was used to evaluate the individual’s attention to positive and negative information in their life. Relative negative attention bias is expressed as ANI/API. The scale is scored on a 5-point scale, with 1 meaning “not at all” and 5 meaning “completely conform.” The sum of the items corresponding to each factor is the factor score. The higher the API score, the greater the attentional bias to positive information, and the higher the score of ANI, the greater the attentional bias to negative information. The Cronbach’s alpha for the API and ANI scales was 0.86 and 0.82, respectively. The test–retest reliability of API is 0.79, and ANI 0.62.

#### Eysenck personality questionnaire

The Eysenck Personality Questionnaire (EPQ) was compiled by Professor Eysenck and the revision committee was chaired by Professor Yaoxian Gong. Gong conducted the standardization of the Chinese version of the EPQ questionnaire in 1983. The revised EPQ for children measures four factors (introversion, neuroticism, psychoticism, and masking) across 88 items with yes-or-no answer choices. The Chinese version of the EPQ has good reliability and validity owing to working standardization. The correlation coefficient of EPQ scale ranges from 0.83 to 0.90, with a Cronbach’s alpha ranging from 0.68 to 0.81. According to the formula in the EPQ scale, the division between neuroticism and non-neuroticism is calculated using the formula *M* ± 0.67 *SD*, or 12.31 + 0.67 ^*^ 4 = 14.99. People with scores higher than 14.99 points are considered to experience neuroticism (Gong, 1983).

#### Zung self-rating depressive symptoms scale

The Zung Self-Rating Depressive Symptoms Scale (SDS) was developed by William W. K. Zung in 1965 and translated into Chinese by Wang et al. (1999). Participants assessed their emotional state in the previous week. The SDS consists of 20 questions with each answered on a 4-point scale with 1 meaning “never or occasionally,” and 4 meaning “always.” The total points scored act as the raw score, and the statistical result is based on the standard score. The raw score needed to be multiplied by 1.25 and rounded up to an integer to be the standard score. A standard score above 52 indicates the presence of depressive symptoms, while a score below or equal to 52 indicates the absence of depressive symptoms. The Cronbach’s alpha of SDS in this study was 0.861.

### Statistical analyses

**Figure 1:**
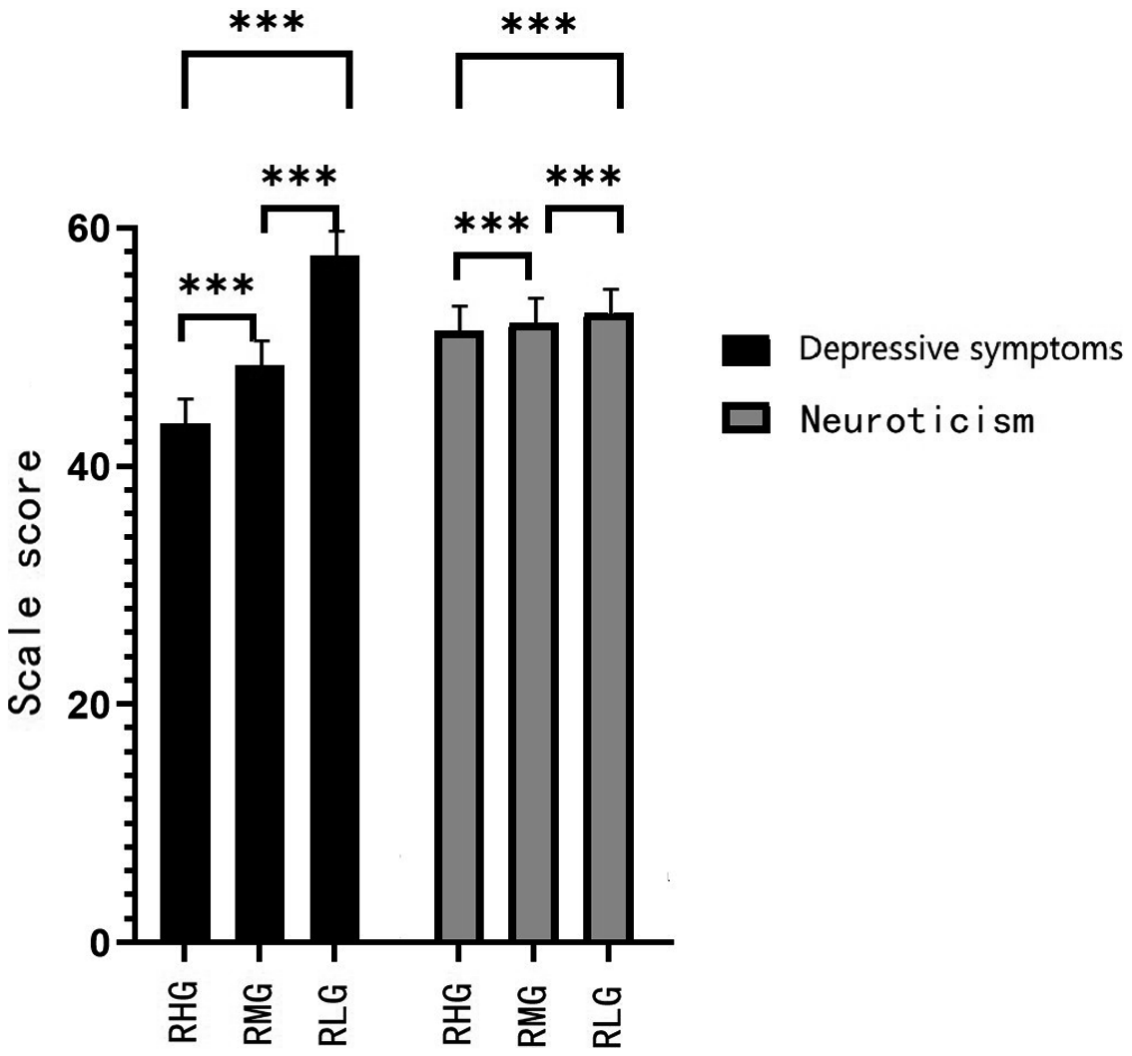
Comparison of scores on depressive symptoms and neuroticism in RHG, RMG and RLG. Data are expressed as means ± standard error of the mean (SEM). ^***^ *p* < 0.001.

**Figure 2:**
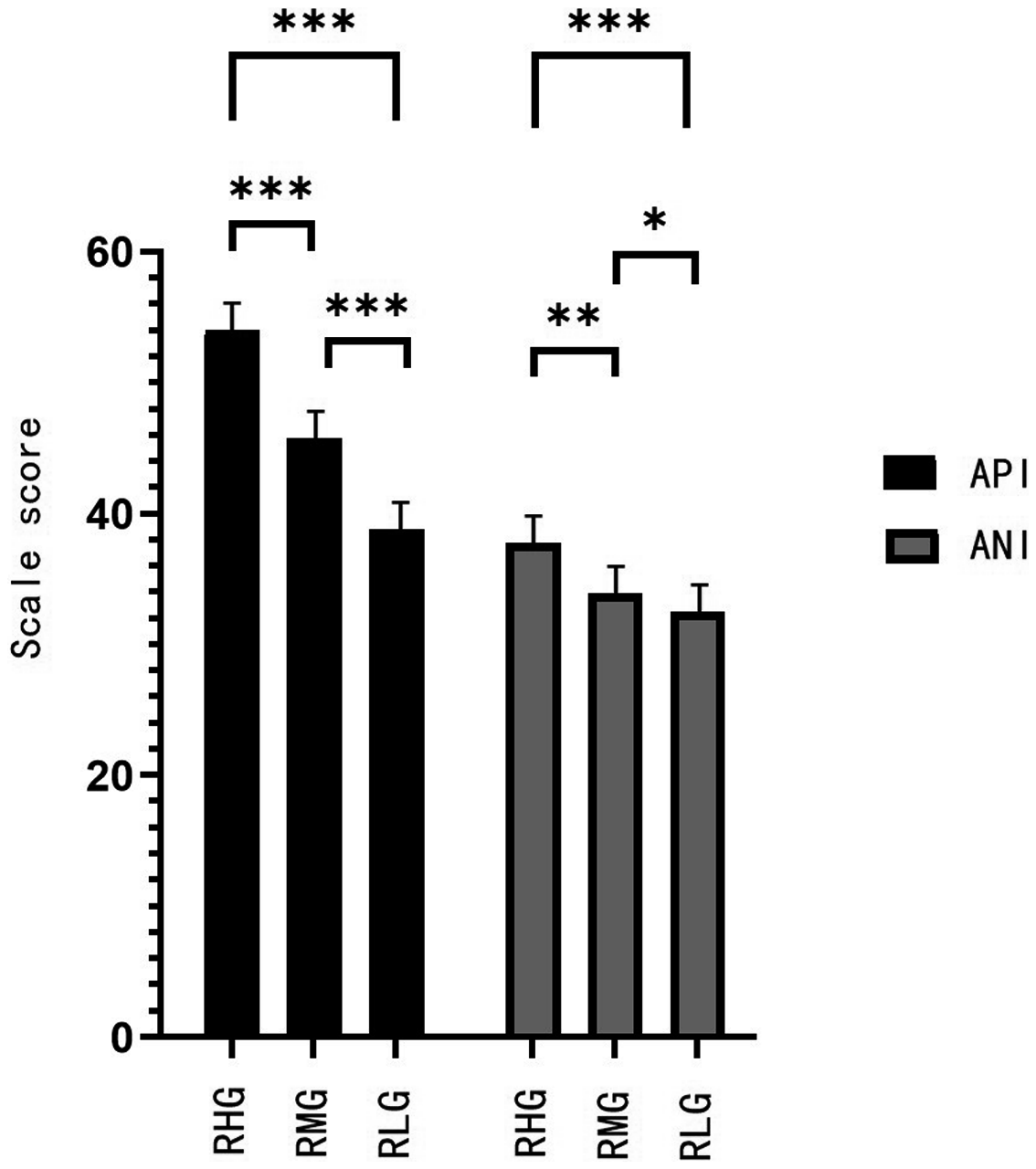
Comparison of scores on API and ANI factors in RHG, RMG and RLG. Data are expressed as means ± standard error of the mean (SEM). ^*^ *p* < 0.05, ^**^ *p* < 0.01，^***^ *p* < 0.001.

**Figure 3:**
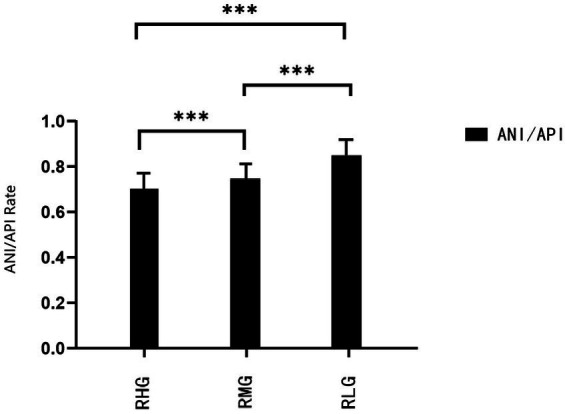
Comparison of ANI/API ratio in RHG, RMG, and RLG. Data are expressed as means ± standard error of the mean (SEM). ^***^ *p* < 0.001.

**Figure 4:**
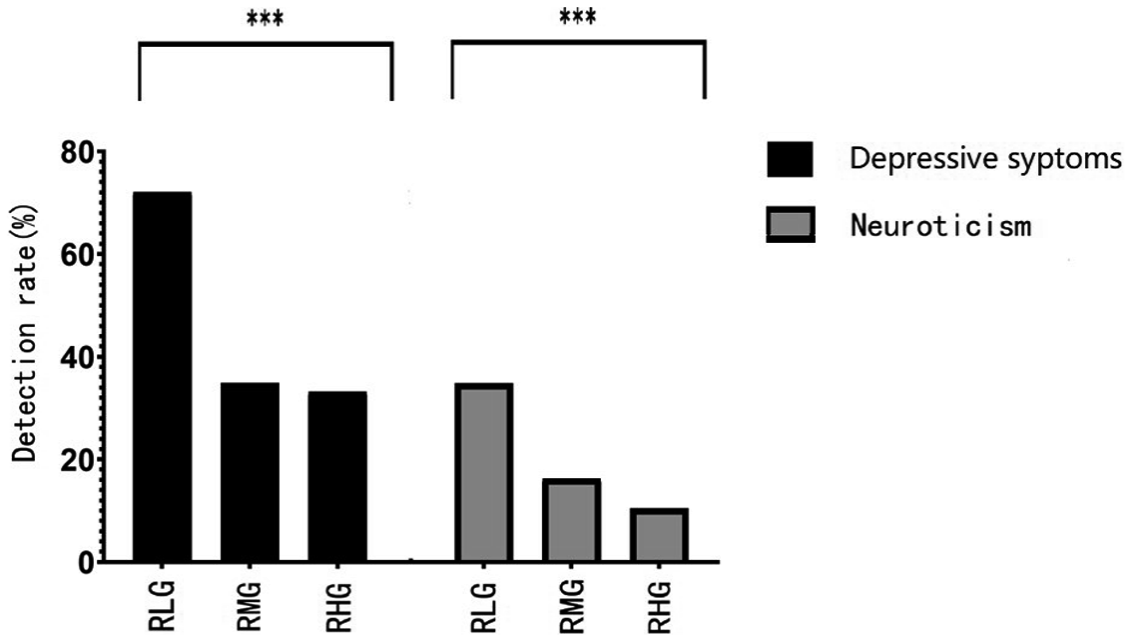
Comparison of the detection rate of depressive symptoms and neuroticism in RHG, RMG, and RLG. Data are expressed as percentage. ^***^ *p* < 0.001.

The collected data were analyzed using SPSS 21.0. The statistical data of measurement statistics were analyzed using *t-*tests, analysis of variance (ANOVA), and non-parametric tests. (Figures 1–3; Tables 1, 2). The statistical data of count statistics were analyzed using a Chi-square test (Figure 4). Cohen’s d and effect size (*r*) were conducted to determine the effect size between the two groups (Table 1), and the between variables were correlated using Pearson’s correlation analysis (Tables 3, 4). Additionally, multiple linear regression analysis was used to analyze the relationship between neuroticism, attentional bias, resilience, and depressive symptoms. Depressive symptoms (SDS) scores were used as dependent variables, while neuroticism, attentional bias, and resilience factors were used as independent variables. Variables were screened using the stepwise method, and factors affecting depressive symptoms were controlled as co-variables (Table 5). Significance levels were set at 0.05 and all tests were two-sided.

**Table 1:** Comparison of the resilience scores of RHG, RMG, and RLG (Mean ± SD).

	RLG	RMG	RMG	RHG	RLG	RHG
Number	190(12.7%)	1,064(71.3%)	1,064(71.3%)	239(16%)	190(12.7%)	239(16%)
Mean ± SD	40.43 ± 10.522	64.57 ± 8.946	64.57 ± 8.946	92.82 ± 5.749	40.43 ± 10.522	92.82 ± 5.749
K-W	−627		−1278.5		−651.5	
P	0.000		0.000		0.000	
Cohen’s d	−2.472		−3.757		−6.179	
Effect Size(r)	−0.777		−0.883		−0.951	

**Table 2:** Comparison of the Depressive symptoms, Neuroticism, API, ANI, and ANI/API scores of RHG, RMG, and RLG (Mean ± SD).

Groups	Depressive symptoms	Neuroticism	API	ANI	ANI/API
RLG	57.638 ± 8.481	52.586 ± 1.280	38.811 ± 7.520	32.516 ± 6.295	0.848 ± 0.141
RMG	48.435 ± 10.226	52.036 ± 1.107	45.746 ± 5.726	33.927 ± 5.301	0.747 ± 0.113
RHG	43.572 ± 13.477	51.408 ± 1.228	53.962 ± 6.885	37.766 ± 9.383	0.702 ± 0.155
RLG/RMG	10.887^***^	6.334^***^	12.209^***^	2.499^*^	9.725^***^
RMG/RHG	5.763^***^	7.720^***^	15.851^***^	3.482^**^	5.706^***^
RLG/RHG	9.972^***^	9.329^***^	15.458^***^	6.820^***^	9.424^***^

^*^ *p* < 0.05; ^**^ *p* < 0.01; ^***^ *p* < 0.001.

**Table 3:** Comparison of depressive symptoms, neuroticism, attentional bias and resilience scores in men and women (Mean ± SD).

	Depressive symptoms	Neuroticism	API	ANI	ANI/API	Tenacity	Optimism	Strength	Resilience
Total									
Male	49.278 ±11.830	51.892 ±1.275	46.306 ±8.050	35.062 ±6.910	0.766 ±0.129	35.89 ±10.000	10.46 ±3.090	23.31 ±6.001	69.66 ±18.233
Female	48.551 ±10.908	52.075 ±1.134	46.100 ±6.982	33.932 ±6.103	0.745 ±1.131	32.53 ±8.705	9.43 ±2.567	21.82 ±5.001	63.78 ±15.131
t	1.187	−2.792	0.504	3.204	3.040	6.621	6.621	4.944	6.437
P	0.236	0.005	0.614	0.001	0.002	0.000	0.000	0.000	0.000

**Table 4:** Correlation analysis of depressive symptoms and resilience, neuroticism, and attentional bias in research group.

Correlation	1	2	3	4	5	6	7
1.Depressive symptoms	1						
2.Neuroticism	0.175^**^	1					
3.ANI/API	0.543^***^	0.199^**^	1				
4.Tenacity	−0.358^***^	−0.295^***^	−0.306^***^	1			
5.Strength	−0.432^***^	−0.286^***^	−0.349^***^	0.881^***^	1		
6.Optimism	−0.327^***^	−0.227^**^	−0.299^**^	0.723^***^	0.746^***^	1	
7.Resilience	−0.399^***^	−0.298^***^	−0.338^***^	0.975^***^	0.951^***^	0.821^***^	1

** *p* < 0.01;

*** *p* < 0.001.

**Table 5:** Correlation analysis of depressive symptoms, neuroticism, attentional bias, and resilience in RHG, RMG, RLG, male, and female.

Depressive symptoms	Neuroticism	API	ANI	ANI/API	Tenacity	Optimism	Strength	Resilience
RHG	−0.115	−0.042	0.469^***^	0.534^***^	0.201^**^	0.001	−0.017	0.119
RMG	0.148^*^	−0.382^***^	0.184^**^	0.502^***^	−0.259^***^	−0.176^**^	−0.386^***^	−0.337^***^
RLG	0.228^**^	−0.376^***^	−0.081	0.369^***^	−0.227^**^	−0.318^***^	−0.272^**^	−0.239^**^
Male	0.083^*^	−0.378^***^	0.122^*^	0.533^***^	−0.354^***^	−0.291^***^	−0.413^***^	−0.379^***^
Female	0.248^**^	−0.438^***^	0.175^**^	0.549^***^	−0.381^***^	−0.376^***^	−0.463^***^	−0.436^***^

* *p* < 0.05;

** *p* < 0.01;

*** *p* < 0.001.

## Results

### Resilience subgroups

The average scores of resilience were calculated and categorized. Participants who scored higher than the average score plus 1 standard deviation were assigned to the resilience high-score group (RHG); those who scored between the average score plus or minus 1 standard deviation were assigned to the resilience medium-score group (RMG); and those who scored lower than the average score minus 1 standard deviation were assigned to the resilience low-score group (RLG). The average score on resilience and the standard deviation of the research group was 66.02 ± 16.621. The RLG, RMG, and RHG delimitation scores were 49.399 and 82.641. A total of 239 (16%) participants were in the RHG, 1,064 (71.3%) participants were in the RMG, and 190 (12.7%) participants were in the RLG. Non-parametric test showed a statistically significant difference in the resilience score of RLG, RMG, and RHG (K-W = 943.305, *p* < 0.001). Significant differences were also found between the RLG, RMG, and RHG groups in a two-way comparison: RLG and RMG (Cohen’s d = −2.472, *r* = −0.777); RMG and RHG (Cohen’s d = −3.757, *r* = −0.883); and RLG and RHG (Cohen’s d = −6.179, *r* = −0.951). Table 1 illustrates these findings.

### Comparison of depressive symptoms, neuroticism, attentional bias of RHG, RMG, and RLG

The depressive symptoms, neuroticism, API and ANI, and ANI/API ratio had significant statistical differences among the RHG, RMG, and RLG (*p* < 0.05; Figures 1–3; Table 2).

### Comparison of the detection rate of depressive symptoms and neuroticism proclivity among RHG, RMG, and RLG

A total of 585 participants with depressive symptoms were detected, with a detection rate of 39.2%. Depressive symptoms accounted for only 32.2% in the RHG, 34.9% in the RMG, and 72.1% in the RLG (*χ^2^* = 99.597, *p* < 0.001). Neuroticism accounted for 10.5% in the RHG, 16.3% in the RMG, and 35.8% in the RLG (*χ^2^* = 52.511, *p* < 0.001). The detection rates of depressive symptoms and neuroticism in the Chi-square statistical analysis results are shown in Figure 4.

### Comparison of average scores of depressive symptoms, neuroticism, attentional bias, and resilience between men and women in the research group

The survey showed that 252 (44.4%) of men had depressive symptoms and 333 (36%) of women had depressive symptoms. There was a significant difference between the men and women (χ^2^ = 10.336, *p* < 0.01), but no significant difference in the average scores of depressive symptoms and API between gender. The total and factor scores of resilience, ANI, and ANI/API were significantly higher in men than in women (*p* < 0.01), and the neuroticism score for women was significantly higher than that of men (*p* < 0.01) as shown in Table 3.

### Correlation analysis of depressive symptoms, neuroticism, attentional bias, and resilience in the research group

Depressive symptoms had a very significant positive correlation with neuroticism and ANI/API ratio (*r* = 0.175–0.543, *p* < 0.01), with resilience and its 3 dimensions had a significant negative correlation (*r* = 0.327–0.432, *p* < 0.001). Neuroticism had a very significant positive correlation with the ANI/API ratio (*r* = 0.199, *p* < 0.01), and a very significant negative correlation with resilience and its 3 dimensions (*r* = 0.227–0.295, *p* < 0.01). Resilience had a very significant positive correlation with its 3 dimensions (*r* = 0.821–0.975, *p* < 0.001). Table 4 further illustrates these results.

### Correlation analysis of depressive symptoms and neuroticism, attentional bias, and resilience in RHG, RMG, RLG, male, and female

According to the Pearson’s correlation analysis, the depressive symptoms of the RHG had a significant positive correlation with ANI, ANI/API ratio, and tenacity (*p* < 0.01). Depressive symptoms in the RMG had a significant positive correlation with neuroticism, ANI, and ANI/API ratio (*p* < 0.01), and a significant negative correlation with API, resilience, and its three factors tenacity, strength, and optimism (*p* < 0.01). The depressive symptoms of the RLG had a very significant positive correlation with neuroticism and ANI/API ratio (*p* < 0.01) and a very significant negative correlation with API, and resilience across its three factors of tenacity, strength, and optimism (*p* < 0.01). Male and female depressive symptoms were significantly positively correlated with neuroticism and ANI (*p* < 0.05), and negatively correlated with resilience, its three dimensions, and API (*p* < 0.001; Table 5).

### Multiple linear regression analysis between neuroticism, attentional bias, resilience, and depressive symptoms in the research group

Depressive symptoms (SDS) scores were used as dependent variables, while neuroticism, attentional bias, and resilience factors were used as independent variables. We included gender, age, ethnicity, neuroticism, attentional bias, and psychological resilience as independent variables in the linear regression analysis and entered the equation by stepwise method. The results showed that gender, age, ethnicity, neuroticism, and optimism did not enter the equation and had no significant effect on depressive symptoms in Chinese college students. API (*B* = − 0.752, standardized β = −0.046, *p* < 0.001), A NI (*B* = 0.796, standardized β = −0.042, *p* < 0.001), tenacity (*B* = 0.139, standardized β = −0.053, *p* < 0.01), and strength (*B* = −0.691, standardized β = −0.093, *p* < 0.001) entered the equation and these variables had a more significant effect on depressive symptoms in Chinese college students (Table 6).

**Table 6:** Multiple linear regression analysis between attentional bias, resilience, and depressive symptoms in the research group.

	B	Standardized β	t-value	P	95%CL	
API	−0.752	0.046	−16.360	0.000	−0.842	−0.662
ANI	0.796	0.042	19.147	0.000	0.714	0.877
Tenacity	0.139	0.053	2.627	0.009	0.035	0.243
Strength	−0.691	0.093	−7.419	0.000	−0.873	−0.508

## Discussion

In this study, we aimed to determine the influence of neuroticism, attentional bias, and varying degrees of resilience on the occurrence of depressive symptoms in a sample of Chinese freshman students. The detection rate of depressive symptoms in this study was 39.2%. Online surveys may not require as much disclosure of respondents’ information and may therefore better reflect the actual situation of the respondents. Our findings indicate a high depressive symptom rate among college freshmen, and that depressive symptoms have become a problem requiring urgent attention. This is similar to the results of studies during the COVID-19 pandemic (e.g., Luo et al., 2021). The impact of the COVID-19 pandemic on Chinese college students is multifaceted. For example, 67.65% of college students felt the negative effect of the COVID-19 pandemic on their studies, 51.47% thought that the COVID-19 pandemic would bring employment pressure. Furthermore, 32.35% of college students thought that their families expected too much of them, and the impact of the epidemic on the employment environment at home and abroad made them unable to fulfill their parents’ wishes (Feng and Feng, 2021). In East Asian countries, parents have higher expectations for their children’s academic performance. This cultural emphasis on academic achievement drives students to place great importance on learning knowledge at the expense of developing various competencies, resulting in poor coping skills and a tendency to develop mental health problems (Sun et al., 2013; Yoon et al., 2017). Additional studies have shown that the depressive symptoms of college students are more likely to be affected by factors such as poverty in graduation year, family poverty, dissatisfaction with their major, poor employment prospects, single-parent families, and national minority (Wang M. et al., 2020; Feng et al., 2022). Regarding college students’ personality traits, studies have shown that extroversion, agreeableness, conscientiousness, and openness are negatively correlated with depression, while neuroticism is positively correlated with depression (Han, 2017). This suggests that the personality traits of the college students who participated in this study are one of the factors that cannot be ignored in the formation of their depressive symptoms. During the COVID-19 pandemic, the above factors may have been more pronounced in the development of depressive symptoms in college students.

Why do some people develop depressive symptoms whereas others do not when confronted with similar stress? Resilience is defined as the capacity of an individual to respond to stress and trauma (Martinez et al., 2021). Studies have shown that college students’ resilience is negatively correlated with the degree of depressive symptoms. High resilience can prevent college students from becoming depressed (Wei, 2019) and the preventive effect of resilience has been shown to improve mental health (Akeman et al., 2020). Neuroticism and attentional bias are closely related to depressive symptoms (Kendler et al., 2004; Dai et al., 2019). What are the effects of neuroticism, attention bias, and different degrees of resilience on depressive symptoms?

The rates of depressive symptoms and neuroticism in groups with different degrees of resilience are especially interesting. Depressive symptoms only accounted for 32.2% in the RHG and 34.9% in the RMG, versus 72.1% in the RLG. Neuroticism accounted for 10.5% in RHG, 16.3% in RMG, and 35.8% in RLG. The lower the resilience, the lower the average scores of attention to positive and negative information, and the higher the ANI/API ratio. That is, the lower the resilience, the more likely the attentional bias is to shift to negative information attention. This may be the reason for the higher average scores of depressive symptoms and neuroticism and a higher detection rate of both. The detection rate of depressive symptoms in the RLG was 2.2 times of the RHG, and 2.1 times of the RMG, and the detection rate of neuroticism was 3.4 times of RHG and 2.2 times of the RMG. This implies that low resilience, high neuroticism, and relative attentional bias to negative information are risk factors for the occurrence of depressive symptoms. This result is consistent with previous studies (Kendler et al., 2004; Dai et al., 2019; Wilson and Howard, 2020).

However, are these three factors independent influencing factors in the occurrence of depressive symptoms, or are they interrelated? How relevant are they? The pathological mechanism is not yet clear. Relevant analysis of this research group showed that resilience was significantly negatively correlated with depressive symptoms, neuroticism, and ANI/API; neuroticism was significantly positively correlated with ANI/API and depressive symptoms; and ANI/API was significantly positively correlated with depressive symptoms. The lower the resilience, the higher the relatively negative information attention bias and neuroticism depressive symptoms. Their interaction with depressive symptoms forms a circular effect. Such a vicious cycle may be one of the psychological mechanisms by which depressive symptoms occur (Wang et al., 2019; Sheng et al., 2022).

What are the differences in the correlation of depressive symptoms and neuroticism and attention bias among different resilience groups? According to the results of this study, there were significant differences in depressive symptoms, neuroticism, and attentional bias in the resilience groups, which means the higher the average score on attentional bias to negative information, neuroticism, ANI/API ratio, and tenacity, the more severe the depressive symptoms. Meanwhile, the total score on neuroticism, attention to positive information, and depressive symptoms did not have a significant correlation in the RHG. This indicates that the enhanced attentional bias to negative information and increased ANI/API ratio are the factors susceptible to depressive symptoms in the RHG. In the RHG, that is not the tenacity factor of resilience the higher the better, because each thing has two sides. Over-tenacity and the relatively negative information attention bias may increase the risk of depressive symptoms in the RHG. In the RMG and RLG, higher neuroticism, relative attentional bias to negative information, and a lower degree of resilience and attentional bias to positive information will increase depressive symptom scores. When grouping according to different degrees of resilience, we found varied correlations between depressive symptoms and neuroticism. For the RHG, neuroticism may not have a big impact on depressive symptoms, while for the RMG and RLG, the higher the propensity for neuroticism, the easier it is to fall into depressive symptoms, which is in accordance with previous studies (Kendler et al., 2004) and indicates a close relationship between the degree of neuroticism and resilience. According to the gender grouping in RESEARCH GROUP, men accounted for 38%, while women accounted for 62%, and the detection rate of depressive symptoms in men accounted for 44.4%, which was significantly higher than that in women (36.0%). However, there was no difference in depressive symptoms and API scores between men and women. Men had significantly higher ANI, ANI/API ratio, and resilience than women, and women had significantly higher neuroticism scores than men, indicating that the high detection rate of male depressive symptoms may be due to the greater impact of negative information attention bias, while female neuroticism and psychological elasticity may have a greater impact on depressive symptoms. The correlation analysis between male and female neuroticism, resilience, attention bias, and depressive symptoms shows that the correlation mechanism between these factors and depressive symptoms is almost the same between men and women.

However, which of these factors has a more direct impact on the occurrence of depressive symptoms? The results of multiple regression analysis show that both high ANI and the decrease of API had a significant impact on the occurrence of depressive symptoms. Under normal circumstances, there may be a balance between API and ANI. The decrease of API and the increase of ANI may break that balance, lead to the relative or absolute increase of ANI, and further lead to an increase in depressive symptoms or symptom severity. This proves our previous assumption of using ANI/API to represent the relative degree of ANI. To our surprise, high tenacity and low strength have a significant impact on the occurrence of depressive symptoms, rather than low tenacity and low strength. Low strength may reduce resilience and lead to depressive symptoms. Why high tenacity promotes depressive symptoms should be further investigated in future research. Neuroticism, a relatively high attentional bias to negative information (high ANI/API ratio), and low resilience may increase the risk of depressive symptoms. Neuroticism, resilience, and attentional bias seem to be sensitive psychological indicators for predicting the risk of depressive symptoms. Once stress factors are encountered, low resilience, high neuroticism, and negative attentional bias make it more difficult to recover from depressive symptoms. This explains why some people recover from a stressful situation quickly, while others experience lasting depressive symptoms. This study confirmed that low resilience, neuroticism, and negative attentional bias were not only independent influence factors on depressive symptoms, but also significantly correlated with each other to promote the degree of depressive symptoms. These results confirmed our hypothesis. If we can intervene in these factors and improve their function, it may be an effective way to alleviate depressive symptoms. Increasing resilience and reducing the proclivity of neurotic behavior and attentional bias to positive information are some of the most important measures to improve strategies against stress and prevent and treat depressive symptoms. Studies show that focusing on working out solutions and paying more attention to positive aspects after a traumatic incident may alleviate post-traumatic stress disorder symptoms and increase overall mental health (Alamdar et al., 2020). Our regression analysis showed that tenacity, strength, and positive–negative attentional information bias played the most prominent roles in depressive symptoms, while neuroticism did not play a significant role in the regression analysis. This shows that interventions for depressive symptoms should focus more on attentional bias and psychological resilience. One way is to increase attentional bias to positive information through training, which may lower the relative attention bias to negative information, ANI/API ratio, and degree of neuroticism, thus increasing resilience and lowering the degree of depressive symptoms. This may be more important for male college freshmen, however, since female college freshmen had lower resilience and higher neuroticism in this study. Targeted training and improving resilience may be more effective in preventing and treating depressive symptoms in female freshmen. Future interventions should place great importance on the issues of resilience, attention bias, and neuroticism. Future research should investigate how to improve countermeasures to these problems and implement them for university students of all genders.

### Limitations

Our results may be of great significance for the prevention and treatment of depressive symptoms in college students; however, during the COVID-19 pandemic, Chinese college students’ resilience, neuroticism, and attentional bias may differ according to their personality, family, ethnicity, and academic performance, thus influencing the onset of depressive symptoms. This has not been discussed in depth in this paper due to space limitation. Our research group was limited to college freshmen in Chinese universities and did not cover all grades. Therefore, the findings do not represent the entire college student population, which future research should address in multiple regions.

## Data availability statement

The raw data supporting the conclusions of this article will be made available by the authors, without undue reservation.

## Author contributions

All authors listed have made a substantial, direct, and intellectual contribution to the work and approved it for publication.

## Funding

This study was supported by the Okamoto Memorial Foundation for mental health in Japan (Leiwa 2-year research 1).

## Conflict of interest

The authors declare that the research was conducted in the absence of any commercial or financial relationships that could be construed as a potential conflict of interest.

## Publisher’s note

All claims expressed in this article are solely those of the authors and do not necessarily represent those of their affiliated organizations, or those of the publisher, the editors and the reviewers. Any product that may be evaluated in this article, or claim that may be made by its manufacturer, is not guaranteed or endorsed by the publisher.
